# Feasibility and Acceptability of a School-Based Nutrition Education Toolkit: Findings from an Expert Review

**DOI:** 10.3390/ijerph23050630

**Published:** 2026-05-10

**Authors:** Amelia Sullivan, Bryn Kubinsky, Jade McNamara

**Affiliations:** School of Food and Agriculture, University of Maine, Orono, ME 04469, USA; amelia.sullivan@maine.edu (A.S.); bryn.kubinsky@maine.edu (B.K.)

**Keywords:** expert review, toolkit, school nutrition, nutrition education, implementation resource, adolescents, rural health, nutrition security

## Abstract

**Highlights:**

**Public health relevance—How does this work relate to a public health issue?**

- Rural adolescents face structural barriers to healthy food access, contributing to low fruit intake and increased risk of diet-related chronic diseases.
- The Helping Early Adolescents Live Their Healthiest Youth (HEALTHY) Toolkit supports nutrition security among rural adolescents through school-based educational programming centered on frozen-fruit smoothies.

**Public health significance—Why is this work of significance to public health?**

- Schools require practical, evidence-informed resources to support nutrition programming in real-world settings.
- Toolkit development and evaluation support the real-world implementation and adoption of evidence-based programs.

**Public health implications—What are the key implications or messages for practitioners, policy makers and/or researchers in public health?**

- The HEALTHY Toolkit is perceived as a feasible and acceptable resource for implementing school-based nutrition education programming.
- Effective implementation requires partnerships among schools, public health professionals, and community organizations to maximize long-term impact.

**Abstract:**

Helping Early Adolescents Live Their Healthiest Youth (HEALTHY) is a theory-driven, school-based nutrition education program grounded in frozen-fruit smoothie taste tests that has been shown to improve fruit intake and promote nutrition security among rural adolescents. To support broader dissemination, a comprehensive implementation toolkit and website were developed. This study conducted an expert review to evaluate the feasibility, clarity, and acceptability of the HEALTHY Toolkit. A survey collected quantitative (Likert-scale) and qualitative (open-ended) data, which were analyzed using descriptive statistics and content analysis. Experts (*N* = 15) primarily included Registered Dietitians (40%, *n* = 6) and school nutrition professionals (33%, *n* = 5). Experts reported high agreement that the HEALTHY Toolkit provided sufficient detail and guidance for implementation, indicating strong feasibility, clarity, and acceptability. Mean scores across Toolkit sections were high: Part I. Foundations (4.70 ± 0.26), Part II. Program Design and Implementation (4.64 ± 0.41), and Part III. Tools and Resources (4.14 ± 0.17). These findings support the use of formative evaluation approaches, such as expert review, to optimize implementation resources and strengthen readiness before dissemination. Overall, the HEALTHY Toolkit was perceived as a promising resource for supporting the implementation of the HEALTHY program.

## 1. Introduction

Adolescence is a critical period for establishing dietary behaviors that influence both immediate and long-term health outcomes [1,2,3,4,5,6,7]. However, adolescents in the United States (U.S.) exhibit poor diet quality [1,5,8,9,10,11]. Fruit intake, in particular, consistently falls below national recommendations [10,11]. While disparities in diet quality and fruit intake exist among all adolescents in the U.S., they are particularly pronounced in rural communities [12,13,14,15,16]. Structural barriers within food systems, including higher food costs, fewer full-service grocery stores, and limited access to fresh produce, further constrain opportunities to improve diet quality outside the school food environment [12,13,14,15,16,17,18]. Therefore, the school food environment is a key setting in shaping dietary behaviors and overall nutrition security for rural adolescents (i.e., consistent access to nutritious foods that support optimal growth, development, and long-term health) [19,20,21,22].

The National School Lunch Program (NSLP), situated within the school food environment, provides a structured and reliable source of nutrition for millions of adolescents and offers a key opportunity to promote fruit intake [23,24,25]. Although federal meal pattern requirements mandate fruit offerings, persistent challenges remain related to student acceptance and fruit waste [26,27,28,29,30]. These challenges underscore the need for innovative, feasible strategies that promote fruit consumption while aligning with the operational realities of school nutrition programs.

School-based nutrition education programs have demonstrated potential to improve dietary behaviors, particularly when they are engaging, developmentally appropriate, and grounded in behavioral theory [31,32,33,34]. Smoothie-based programming has emerged as a promising strategy to promote fruit intake by aligning with student preferences, offering flexibility in ingredient sourcing, and integrating experiential learning opportunities [35,36,37,38]. However, successful implementation of such programs requires alignment with school infrastructure, staff capacity, NSLP requirements, and curriculum needs.

Implementation toolkits are increasingly used to translate research into practice by providing structured guidance, educational materials, and resources to support program delivery. These toolkits can improve program adoption, consistency, and scalability across settings. However, the development and evaluation of toolkits are not consistently documented in the literature, limiting transparency and reproducibility. Formative evaluation approaches, such as expert review, are critical for assessing feasibility, clarity, and usability of such toolkits prior to dissemination [39]. Expert review can help refine implementation guidance and identify potential barriers to real-world application [39,40,41,42,43,44,45,46].

Helping Early Adolescents Live Their Healthiest Youth (HEALTHY) is a theory-driven, school-based nutrition education program designed to promote fruit intake among rural adolescents through a four-session frozen-fruit smoothie tasting [36,37]. Previous evaluations of the program demonstrated improvements in fruit intake and strengthened nutrition security among rural adolescents through smoothie consumption [36,37]. The HEALTHY smoothie recipes meet several NSLP meal pattern requirements (e.g., fruit, fluid milk, and meat/meat alternative credits) and primarily consist of frozen fruit, low-fat milk, and Greek yogurt. Social Cognitive Theory and Choice Architecture informed the program design. Social Cognitive Theory constructs addressed through program activities include repeated exposure, observational learning via peer modeling, outcome expectations through brief nutrition education, and self-efficacy and self-regulation through goal-setting activities. Choice Architecture principles were incorporated by offering smoothies in a convenient, visually appealing, and highly accessible cafeteria setting. Comprehensive details regarding the program design, educational objectives, nutritional composition of the smoothies, implementation procedures, quasi-experimental outcome evaluations, student-centered process findings, and school nutrition directors’ systems-level perspectives on implementation feasibility and sustainability are published elsewhere and are also readily available on the HEALTHY website [36,37,47,48].

Based on our evaluations of the program and to support broader dissemination, the research team developed a comprehensive HEALTHY Toolkit and a corresponding Google website to guide school nutrition professionals and educators in program delivery. Therefore, the purpose of this study was to conduct an expert review to evaluate the perceived feasibility, clarity, and acceptability of the HEALTHY Toolkit as a resource to support real-world implementation prior to broader dissemination.

## 2. Materials and Methods

### 2.1. Study Design

This study conducted a formative expert review design to evaluate the feasibility, clarity, and acceptability of the HEALTHY Toolkit, a smoothie-based nutrition education resource designed to support school nutrition directors and educators in implementing the HEALTHY program. An anonymous online survey administered via Qualtrics XM (Qualtrics, Provo, UT, USA) captured data from the expert reviewers. Quantitative data were collected using Likert-scale items to assess expert agreement regarding the Toolkit’s feasibility, clarity, and acceptability, while qualitative data were collected through open-ended questions to capture unique, practice-informed feedback from expert reviewers.

This study was reviewed and approved by the University of Maine Institutional Review Board (#2025-10-18, 18 October 2025). An informed consent statement was presented on the first page of the anonymous online survey, and consent was implied upon completion.

### 2.2. Participants and Recruitment

Expert reviewers were purposively sampled based on professional experience in school nutrition, nutrition education, adolescent health, and/or school-based programming through the lead researcher’s professional networks [49]. These networks included academic collaborators, Extension and school nutrition contacts, USDA multistate research group colleagues, and school-based organizations. Eligible participants included school nutrition directors or managers, Registered Dietitians, teachers or health educators, school garden coordinators, FoodCorps service members, school or district administrators, and academic or research faculty with relevant expertise. Snowball sampling was also used, whereby participants were invited to refer additional experts who might be interested in reviewing the HEALTHY Toolkit [50].

Following an initial email invitation, interested individuals received a follow-up email with a detailed study description and a link to the anonymous online survey administered via Qualtrics. Recruitment occurred between 1 December 2025 and 31 January 2026. The target sample size was 15 expert reviewers, consistent with previous studies employing similar methods [39,40,41,42,43,44,45,46]. The survey required approximately 45 to 60 min to complete, and participants had four weeks to respond after receiving the survey link. Upon completion, participants were redirected to a separate survey where they could voluntarily provide an email address to receive a 75-dollar Amazon electronic gift card as an honorarium.

### 2.3. HEALTHY Toolkit Development

Following comprehensive evaluations of the HEALTHY program in two experimental schools in early 2025 [36,37,47,48], the study’s lead researchers developed the HEALTHY Toolkit between August and October 2025 to support the dissemination and implementation of the HEALTHY program. The Toolkit was developed using Canva (Canva Pty Ltd., Sydney, NSW, Australia).

The HEALTHY Toolkit is a 41-page PDF document organized into three sections: (1) Part I. Foundations, (2) Part II. Program Design and Implementation, and (3) Part III. Tools and Resources. Part I. Foundations provides a high-level overview of the HEALTHY program, including the rationale and feasibility for providing smoothies in school settings, the theoretical framework guiding program development, and key findings from the initial program evaluation [36,37,47]. Part II. Program Design and Implementation provides a comprehensive overview of the program structure and step-by-step implementation guidance, including required supplies and equipment, educational objectives, smoothie recipes with nutrient analysis, and student engagement strategies. Part III. Tools and Resources includes guidance on adapting and scaling the program across school settings, along with recommendations for external resources.

In addition to the HEALTHY Toolkit, the research team developed a corresponding website hosted on Google Sites [47]. The website provides a high-level overview of the program, access to the Toolkit, downloadable materials referenced throughout the Toolkit, open access to the supporting research, a curated list of external resources, and contact information for those interested in implementation. The HEALTHY Toolkit and associated resources are available through the program website (see reference [47]).

### 2.4. Expert Review Survey

The expert review survey consisted of five sections and included a total of 67 quantitative and qualitative items, aligned with the HEALTHY Toolkit’s structure and content, as detailed in the Supplementary Material. These items were developed by the researchers based on their subject-matter expertise in school nutrition, program development, and implementation and were further informed by, and compared with, previously published expert review studies [39,40,41,42,43,44,45,46]. The funders had no role in the development of survey items, data analysis, or interpretation of findings.

The survey consisted of five sections and was administered via Qualtrics. The first section included four questions assessing participants’ characteristics (Supplementary Table S1). The first question collected participants’ current role or areas of work, with response options including school garden coordinator, Registered Dietitian, teacher/health educator, school nutrition director or manager, researcher/academic faculty, FoodCorps service member, school or district administrator, or other (please specify), and participants could select all that applied. The second question asked about the highest level of education completed. The third question asked how many years of experience participants had in their current field or area of work (‘less than 1 year,’ ‘1–5 years,’ ‘6–10 years,’ ‘11–15 years,’ ‘16–20 years,’ or ‘more than 20 years’). Finally, one open-ended question asked participants to elaborate on their current work: “In one to two sentences, could you briefly describe your line of work?”

Sections two through four corresponded to the three primary sections of the HEALTHY Toolkit: Part I. Foundations, Part II. Program Design and Implementation, and Part III. Tools and Resources. These sections included several 5-point Likert-scale items assessing agreement (‘strongly disagree’ to ‘strongly agree’) with statements related to feasibility, clarity, and acceptability. In total, sections two through four included 42 quantitative items. Example survey items included the following: “This section clearly conveys the importance of smoothies as a strategy to promote fruit intake,” “The implementation steps are clearly written and easy to follow,” and “The educational resources are easy to access and apply in practice.”

Additionally, at the end of sections two through four, participants were asked the same three open-ended questions: (1) “Please describe any components of this part of the E-Guide that were difficult to follow or could benefit from additional clarifications,” (2) “Is there any additional information or content you think would strengthen this part of the E-Guide? Please explain below,” and (3) “Which aspects of this part of the E-Guide were most helpful or useful to you, and why?” See Supplementary Tables S2–S4 for the respective items.

The final section of the survey addressed overall perceptions of the HEALTHY Toolkit. This section included five 5-point Likert-scale items evaluating perceived program impact and utility (‘strongly disagree’ to ‘strongly agree’), two 10-point sliding-scale items assessing program feasibility (‘not at all’ to ‘extremely’), and five open-ended questions capturing broader feedback on strengths and potential barriers to implementation (see Table 1).

### 2.5. Data Analysis

Because survey items were developed by the research team for the purposes of this study and were not previously validated, internal consistency reliability was assessed using Cronbach’s alpha. Quantitative items used to evaluate Parts II and III demonstrated acceptable internal consistency (α = 0.93 and α = 0.66, respectively), whereas items for Part I demonstrated lower internal consistency (α = 0.53). Despite the lower reliability estimate for Part I, all items were retained because the research team determined that each item represented conceptually important aspects of the Toolkit and contributed to the overall evaluative purpose of the study. All survey items are available in the Supplementary Material.

Quantitative data were analyzed using descriptive statistics in IBM SPSS Statistics for Mac (v29.02.0; IBM Corp., Armonk, NY, USA). The primary analytical aim of the quantitative component was to descriptively assess expert reviewers’ level of agreement regarding items assessing the perceived feasibility, clarity, and acceptability of the three primary Toolkit parts and their respective subcategories. Means and standard deviations were calculated for individual survey items and for aggregated scores within each HEALTHY Toolkit section. Section-level scores were calculated by averaging item responses within each corresponding Toolkit part.

Qualitative data from open-ended survey responses were analyzed using basic content analysis [51]. This approach was selected because the open-ended items were designed to capture unique, practice-informed feedback from expert reviewers rather than to generate an in-depth interpretive dataset. Responses were exported from Qualtrics into a Microsoft Excel spreadsheet (Microsoft Corporation, Redmond, WA, USA) for organization and analysis.

Researchers collaboratively read all responses several times to become familiar with the dataset and begin identifying meaningful text segments. Segments were then condensed while preserving the core message, and descriptive codes were developed to categorize responses. Through iterative discussion and consensus among the research team, codes were organized into two final categories: (1) strengths and (2) opportunities for improvement.

Following the initial categorization, the research team re-reviewed all response segments and category assignments to resolve discrepancies, refine category definitions, and ensure consistency of interpretation. Reflective discussions were used throughout the process to consider potential researcher bias and the influence of prior subject-matter expertise on data interpretation. Representative response excerpts were retained within each category to support reporting of findings and are described in the context of the three Toolkit parts.

## 3. Results

### 3.1. Participant Characteristics

Fifteen experts participated in the expert review of the HEALTHY Toolkit. The majority were Registered Dietitians (40%, *n* = 6) and school nutrition directors or managers (33%, *n* = 5). Fifty percent of participants held a master’s degree, and 60% had 16 or more years of experience in their field. Additional participant characteristics are presented in Table 2.

### 3.2. Expert Opinion per HEALTHY Toolkit Section

Overall, experts reported high agreement that the HEALTHY Toolkit provided sufficient detail and guidance for implementation. Among the three sections of the HEALTHY Toolkit, Part I. Foundations had the highest overall mean score (4.72 ± 0.26), indicating strong agreement that the program design, theoretical framing, and evidence supporting fruit intake were clearly described.

Part II. Program Design and Implementation received the second highest overall rating (4.64 ± 0.41). Experts generally agreed that this section provided sufficient detail to support the implementation of the HEALTHY program. Within this section, the ‘Program Overview’ (4.87 ± 0.35) and ‘Education Overview’ (4.73 ± 0.33) subsections received the highest ratings. In contrast, the ‘HEALTHY in Action’ subsection received the lowest rating (4.38 ± 0.67).

Part III. Tools and Resources had the lowest overall mean score among the three sections (4.14 ± 0.17), although ratings remained within the agreement range of the Likert scale (i.e., ≥4.0 indicating agreement). Experts agreed that the strategies provided were practical and useful for adapting the program across school settings (see Table 3).

#### 3.2.1. Part I—Foundations

Qualitative feedback highlighted several strengths of Part I. Foundations, particularly the explanation of how smoothies fit within school meal programs and the rationale for implementation. Reviewers noted: (1) “I feel like the section on increased literacy and benefits outside of the nutritional focus are a huge sell and will be a huge benefit for this guidebook,” (2) “I think the ‘Impact’ section was the most interesting and would be most useful to the school nutrition department when considering if they want to start a smoothie station/program,” and (3) “I loved the section on how smoothies fit into school meals! This was very well researched, and I appreciated that the context of where these smoothies might be going was taken into consideration.”

Reviewers also emphasized the importance of the crediting information, with comments such as: (1) “The ‘see our quick tips’ for crediting smoothies’ section I found most helpful,” and (2) “It was very smart to add tips to crediting smoothies, and your chart and information are very organized and comprehensive.” Experts also appreciated the organization and clarity of the content, with one reviewer stating: “I like how organized the information is, how attractive to look at and read it is. It is informative, easy to understand, clear, and concise.”

Experts also identified areas where additional clarification could strengthen this section. For example, reviewers suggested that clearer guidance on implementation contexts would be helpful, stating: “Clarifying where exactly you see this program fitting into school meals—breakfast, lunch, snack, in the classroom, all of them?” This was echoed by another reviewer, who stated: “A sample menu on how smoothies can work into school lunch menus.” Additional feedback suggested that further clarification regarding crediting could strengthen this part of the Toolkit. Some reviewers noted: (1) “I think a visual of a smoothie with the ingredients inside would be engaging and then you could point to the credits it meets,” and (2) “A good graphic to add might be a graphic with a smoothie recipe (single serving) and how it credits toward a meal.”

#### 3.2.2. Part II—Program Design and Implementation

Qualitative feedback highlighted the clarity and practicality of the implementation guidance in Part II of the Toolkit. One reviewer stated: “The implementation steps were clear and well organized!” Another noted: “This is an easy read that is clear, and goals are outlined very well.” Experts also valued the detailed equipment and supply guidance, with one reviewer commenting: “The detailed list of supplies and equipment was very helpful. I think schools would be looking for this level of detail, so they know exactly what they need.” This was echoed by another reviewer who stated: “I also love the different options for equipment that you include.”

Reviewers also noted the importance of student engagement strategies included in the Toolkit. Reviewers wrote: (1) “YAY for student leadership being built into the program—so important and a great part of making this effective!” and (2) “The student engagement strategies seemed feasible and attractive.” Another reviewer highlighted the program’s theoretical grounding, stating: “By pairing skill-building through education (Social Cognitive Theory) with an environment that nudges healthier behavior (Choice Architecture), the HEALTHY program reinforces fruit consumption.”

Others expressed positive perceptions of the SMART (Specific, Measurable, Achievable, Relevant, and Time-bound) goals section, stating: “I personally found the SMART Goals section and graphic to be helpful. Trying to explain what each SMART letter means can be difficult, but I really like how it’s explained here! I’m going to steal this for my own use!” In contrast, some experts indicated that the discussion of the SMART goals materials could be simplified. For example, one reviewer wrote: “‘SMART Goals’ (pg. 25): It could be helpful to add a brief script for how teachers or nutrition staff could introduce what SMART goals are. Just a skeleton of ‘what and why so that they can easily explain to students.’” Another noted: “I think the SMART goals need more differentiation. Maybe provide a few example goals for students to initially select from until they become more familiar with the program and how it can benefit them health-wise.”

Reviewers also provided accessibility suggestions. For example, the following suggestion was provided: “I think the curriculum resources (i.e., the smoothie posters, recipe cards, SMART goals, and other printable material) could be improved upon by providing translated versions (or linking quality translation sites).” Finally, reviewers provided suggestions related to feasibility and implementation, including: (1) “Reimbursement was mentioned—this will be a huge factor in many states,” (2) “Roughly how much time would be needed to implement HEALTHY? I think schools would be looking for this information, even if it’s just an estimate, and it might vary. Time is always on schools’ minds, and how to maximize the use of time,” and (3) “I would suggest including ways to collaborate with food service staff to implement this program.”

#### 3.2.3. Part III—Tools and Resources

Qualitative feedback highlighted the usability of Part III of the Toolkit and the practicality of the resources provided. One reviewer commented: “I thought this [part] was easy to navigate and understand,” while another echoed this sentiment: “This section is well written and will be pivotal in the success of this program.”

Other reviewers emphasized the value of the scalability guidance: (1) “Thank you for the ‘Scalable Strategies,’ that is usually the worst part of making items in large quantities,” (2) “I truly liked the scalability of all the recipes,” and (3) “I think the scalable strategies are the most helpful; it seems like it would save a lot of time with ordering.”

Experts also appreciated the inclusion of the external resources and evaluation tools, with one reviewer noting: “I also like the evaluation tools section—good idea to have students reflect after they do the event,” emphasized by another stating: “I liked the simple evaluation tool—I think it’s practical for most schools to implement. I also liked that you could click the button to access all the resources. That was a nice touch!”

Some reviewers also identified opportunities for refinement within this section. One reviewer noted that the student evaluation may be difficult to complete in practice, stating: “The evaluation may be too long for the students to complete.” Another reviewer expanded on this suggestion: “I would suggest a downloadable Google evaluation form instead of having students complete a written survey.” Another area identified for improvement was the inclusion of cost-related information. One reviewer noted: “Scalable Strategies—this is not super fun, but adding a cost analysis of how much a batch of smoothies would cost could be helpful to see how realistically a school could implement this.”

### 3.3. Expert Opinion Regarding HEALTHY Toolkit Key Objectives

Overall, expert reviewers strongly agreed that the HEALTHY Toolkit has the potential to improve fruit intake, increase nutrition knowledge, and serve as a feasible program in school settings. See Table 4 for additional details.

## 4. Discussion

This study conducted a formative expert review to evaluate the feasibility, clarity, and acceptability of the HEALTHY Toolkit as a resource to support real-world implementation prior to broader dissemination. Overall, expert reviewers reported consistently high levels of agreement across all Toolkit parts, indicating strong perceived feasibility, clarity, acceptability, and overall usefulness for implementation in school settings. Notably, all quantitative ratings remained in the “agree” range or higher, suggesting that the Toolkit is favorably perceived by reviewers and appears well developed and aligned with the needs of school nutrition professionals and educators. Experts also agreed that the Toolkit has the potential to improve fruit intake, increase nutrition knowledge, and support the delivery of experiential, school-based nutrition education.

These findings are consistent with previous research highlighting the importance of formative evaluation during program development [39,40,41,42,43,44,45,46]. Formative approaches, including expert review, are used to refine program materials, strengthen content validity, and improve implementation resources prior to dissemination [39,40,41,42,43,44,45,46]. For example, Potvin et al. reported that the formative assessment of a school-based program led to the development of additional implementation resources and the refinement of activities to better align with the target population [41]. Similarly, in the current study, expert feedback provided additional valuable context to complement the high quantitative ratings. Importantly, the suggested refinements primarily reflected opportunities to enhance clarity, usability, and accessibility rather than fundamental concerns regarding the core content or structure of the HEALTHY Toolkit. In some cases, recommended additions (e.g., SMART goal examples) were already included in later sections of the Toolkit, suggesting that some feedback may reflect a partial review rather than substantive gaps in content. Collectively, these findings indicate that the HEALTHY Toolkit is well developed from the perspective of expert reviewers, with opportunities focused on optimization rather than structural revision.

These findings are also consistent with previous research evaluating implementation toolkits [43,44,45], including a study assessing a food insecurity toolkit for higher education institutions, which similarly found that expert reviewers’ feedback focused on improving usability rather than identifying major limitations [44]. In the present study, the absence of low quantitative ratings, alongside positive and constructive qualitative feedback, further supports the potential of the HEALTHY Toolkit for future real-world implementation testing. Notably, expert feedback did not identify any major concerns regarding feasibility or implementation in the school setting. Instead, suggestions focused on enhancing implementation support, including visual materials, time estimates, and cost considerations. These findings are particularly relevant given the operational constraints of school nutrition programs, where considerations such as staffing, reimbursement, and alignment with federal meal requirements are critical for successful implementation [48,52]. Future adaptations of the Toolkit may also benefit from expanded strategies to address structural barriers to nutrition security, support classroom-based implementation by health educators, and incorporate family or caregiver engagement to reinforce learning beyond the school setting.

Taken together, these findings suggest that the HEALTHY Toolkit is not only acceptable in concept but also perceived as feasible to pilot implement within real-world school settings by experts in the field. Given that expert feedback centered on optimization rather than fundamental changes, substantial revisions to the Toolkit were not indicated during this formative review stage. Instead, these findings support next-phase pilot implementation and evaluation, with future studies focused on real-world use, adoption, and iterative refinement across diverse school settings. Future evaluations would also benefit from using formal Implementation Science frameworks to more systematically assess adoption, fidelity, sustainability, and scalability.

### Strengths & Limitations

This study has several strengths. First, the use of both quantitative ratings and qualitative feedback allowed for a comprehensive formative evaluation of the HEALTHY Toolkit. Second, the expert reviewers represented a range of professional roles relevant to school-based nutrition programming, including Registered Dietitians, school nutrition directors, and educators, many of whom had extensive experience in their respective fields.

Limitations should be considered. Participants were recruited through purposive and snowball sampling from the research team’s professional network, which may have introduced selection bias and contributed to favorable ratings. The high mean scores may also indicate potential ceiling effects, which could have limited the rating scale’s sensitivity to detect more subtle differences in perceptions across the Toolkit parts. Findings reflect expert perceptions of feasibility and potential impact rather than actual implementation outcomes, end-user experiences, or demonstrated effectiveness. Finally, no formal Implementation Science framework was used to structure this formative expert review.

## 5. Conclusions

The HEALTHY Toolkit was perceived by expert reviewers as a feasible, clear, and acceptable resource to support the implementation of a smoothie-based nutrition education program in school settings. The formative expert review provided valuable insights to further optimize and pilot the Toolkit. These findings highlight the importance of incorporating formative evaluation into the development of implementation resources to enhance usability, support real-world applicability, and strengthen implementation readiness.

Given that feedback primarily focused on optimization rather than fundamental changes, the HEALTHY Toolkit is perceived as well-positioned for pilot implementation and broader implementation. Future research should examine implementation and effectiveness across diverse school settings to assess its impact on adolescent dietary behaviors and nutrition security and to further refine the Toolkit based on real-world application. Future Toolkit adaptations should also explore strategies to improve inclusivity, address structural barriers to healthy eating, and engage families and caregivers in supporting adolescent nutrition behaviors.

## Figures and Tables

**Table 1:** Expert review open-ended questions assessing overall opinions of the Helping Early Adolescents Live Their Healthiest Youth (HEALTHY) Toolkit.

Open-Ended Questions	
1	What aspects of the E-Guide overall did you find most helpful or effective?
2	What aspects of the E-Guide overall could be improved or clarified?
3	What potential barriers to implementation do you foresee?
4	Is there anything missing from the E-Guide that you feel should be included?
5	Do you have any additional comments or suggestions about the E-Guide?

**Table 2:** Characteristics of expert reviewers evaluating the Helping Early Adolescents Live Their Healthiest Youth (HEALTHY) Toolkit (*N* = 15).

Variable	% (*n*)
Area of Work	
Registered Dietitian	40 (6)
Teacher/Health Teacher	7 (1)
School Nutrition Director or Manager	33 (5)
FoodCorps Service Member	13 (2)
School or District Administrator	7 (1)
Other	33 (5)
Highest Degree of Education	
Some College or Associate’s Degree	7 (1)
Bachelor’s Degree	43 (6)
Master’s Degree	50 (7)
Years of Experience in Field	
Less than 1 year	7 (1)
1–5 years	7 (1)
6–10 years	13 (2)
11–15 years	13 (2)
16–20 years	7 (1)
More than 20 years	53 (8)

**Notes:** Sample sizes may vary due to missing responses. Under ‘Area of Work,’ reviewers could select multiple options. Regarding the category ‘Other’, responses included: Extension Agent, State Agency, and Retired.

**Table 3:** Agreement per guide parts of expert reviewers evaluating the Helping Early Adolescents Live Their Healthiest Youth (HEALTHY) Toolkit (*N* = 15).

Variable	Mean (Standard Deviation) ^a^
**Part I. Foundations**	**4.72 (0.26)**
**Part II. Program Design and Implementation**	**4.64 (0.41)**
Program Overview	4.87 (0.35)
Implementation Steps	4.67 (0.56)
HEALTHY in Action	4.38 (0.67)
Supplies and Equipment	4.62 (0.49)
Education Overview	4.73 (0.33)
Student Engagement Strategies	4.62 (0.50)
**Part III. Tools and Resources**	**4.14 (0.17)**
Adaptation Strategies	4.71 (0.34)
HEALTHY Educational Resources	3.90 (0.28)
HEALTHY Evaluation Resource	3.92 (0.19)
Additional Resources	4.00 (0.00)

**Notes:** ^a^ Survey response options ranged from strongly disagree (1) to strongly agree (5). HEALTHY: Helping Early Adolescents Live Their Healthiest Youth.

**Table 4:** Agreement of expert reviewers evaluating the Helping Early Adolescents Live Their Healthiest Youth (HEALTHY) Toolkit by Part (*N* = 15).

Variable	Mean (Standard Deviation)
Improve fruit intake among students. ^a^	4.80 (0.41)
Promote the delivery of engaging nutrition education in schools. ^a^	4.87 (0.35)
Increase students’ knowledge and awareness of nutrition. ^a^	4.80 (0.41)
Support school nutrition staff with practical tools for implementation. ^a^	4.67 (0.62)
Enhance collaboration between educators and nutrition professionals. ^a^	4.53 (0.64)
How feasible would it be to use the E-Guide in practice? ^b^	8.62 (1.60)
How confident do you feel that you could implement the program? ^b^	8.67 (2.00)

**Notes:** ^a^ Response options ranged from strongly disagree (1) to strongly agree (5). ^b^ Response options ranged on a sliding scale from not at all (0) to extremely (10).

## Data Availability

Data are available upon reasonable request made to the authors. The data are not publicly available due to privacy and ethical reasons.

## Supplementary Materials

The following supporting information can be downloaded at: https://www.mdpi.com/article/10.3390/ijerph23050630/s1, Table S1: Section One: Survey Items Utilized in an Expert Review Study Assessing the Feasibility and Acceptability of a Smoothie-Based Nutrition Education Implementation Toolkit; Table S2: Section Two: Survey Items Utilized in an Expert Review Study Assessing the Feasibility and Acceptability of a Smoothie-Based Nutrition Education Implementation Toolkit; Table S3: Section Three: Survey Items Utilized in an Expert Review Study Assessing the Feasibility and Acceptability of a Smoothie-Based Nutrition Education Implementation Toolkit; Table S4: Section Four: Survey Items Utilized in an Expert Review Study Assessing the Feasibility and Acceptability of a Smoothie-Based Nutrition Education Implementation Toolkit; Table S5: Section Five: Survey Items Utilized in an Expert Review Study Assessing the Feasibility and Acceptability of a Smoothie-Based Nutrition Education Implementation Toolkit.
