# Supplementary material for: Feasibility and Acceptability of a School-Based Nutrition Education Toolkit: Findings from an Expert Review
Source: Int J Environ Res Public Health. 2026 May 10;23(5):630. doi: 10.3390/ijerph23050630 (PMC13206662; doi:10.3390/ijerph23050630)
Supplement: Supplementary file 1 [file ijerph-23-00630-s001.zip › ijerph-4278083-supplementary.pdf]

Supplementary Materials A. Survey Items

Table S1. *Section One: Survey Items Utilized in an Expert Review Study Assessing the Feasibility and Acceptability of a Smoothie-Based Nutrition Education Implementation Toolkit.*

Survey Items	Response Options
Which of the following best describes your current role or area(s) of work? (Select all that apply.)	(1) School Garden Coordinator; (2) Registered Dietitian; (3) Teacher/Health Educator: (4) School Nutrition Director or Manager; (5) Researcher/Academic Faculty; (6) FoodCorp Service Member; (7) School or District Administrator: (8) Other (please specify)
What is the highest degree or level of education you have completed?	(1) High school diploma or GED; (2) Some college or associate’s degree; (3) Bachelor’s degree (e.g., BS, BA); (4) Master’s degree (e.g., MS, MPH, MEd); (5) Doctoral or professional degree (e.g., PhD, EdD, MD, RD, DNP)
How many years of experience do you have in your current field or area of work?	(1) Less than 1 year; (2) 1–5 years; (3) 6–10 years; (4) 11–15 years; (5) 16–20 years; (6) More than 20 years
In one or two sentences, could you briefly describe your line of work?	Open-Ended

Table S2. *Section Two: Survey Items Utilized in an Expert Review Study Assessing the Feasibility and Acceptability of a Smoothie-Based Nutrition Education Implementation Toolkit.*

Survey Items	Response Options
This section clearly conveys the importance of smoothies as a strategy to promote fruit intake.	(1) Strongly Disagree; (2) Somewhat Disagree; (3) Neither Agree nor Disagree; (4) Somewhat Disagree; (5) Strongly Agree
This section makes smoothies seem like a strategy that school nutrition professionals would be likely to support.	
This section makes smoothies seem feasible to implement in a typical school setting.	
The information presented clearly demonstrates the effectiveness of the intervention and strengthens confidence in using smoothies as a fruit promotion strategy.	
The theoretical framework and rationale are clear and easy to understand.	
Please describe any components of "Part I. Foundations" (pages 1-8) of the E-Guide that were difficult to follow or could benefit from additional clarification.	Open-Ended
Is there any additional information or content you think would strengthen "Part I. Foundations" (pages 1-8) of the E-Guide? Please explain below.	
What aspects of "Part I. Foundations" (pages 1-8) of the E-Guide were most helpful or useful to you, and why?	

Table S3. *Section Three: Survey Items Utilized in an Expert Review Study Assessing the Feasibility and Acceptability of a Smoothie-Based Nutrition Education Implementation Toolkit.*

Survey Items	Response Options
The Program Overview section is clearly written and easy to follow. [a]	(1) Strongly Disagree; (2) Somewhat Disagree; (3) Neither Agree nor Disagree; (4) Somewhat Disagree; (5) Strongly Agree
The purpose and objectives of the program are clearly explained. [a]	
The overview provides adequate context and background to support program implementation. [a]	
The content of the overview aligns logically with the goals presented in Part I. Foundations. [a]	
The implementation steps are clearly written and easy to follow. [b]	
The sequence of steps is logical and well-organized. [b]	
The described steps appear feasible to carry out within a school setting. [b]	
The section provides adequate detail to support successful implementation. [b]	
The examples presented in HEALTHY in Action are relevant, realistic, and practical for school settings. [c]	
This section effectively demonstrates how the E-Guide can be applied in real-world contexts. [c]	
It is clear where and how to access all supplemental materials and references (e.g., on the website). [c]	
The supplies and equipment list is clearly organized and comprehensive. [d]	
The information provided is practical and appropriate for school-based implementation. [d]	
The section offers sufficient guidance on sourcing items and identifying suitable alternatives. [d]	
The overarching themes of these sections are clear and cohesive. [e]	
The educational goals are clearly defined and easy to understand. [e]	
The educational materials and resources are appropriate for the intended audience. [e]	
The Nutrition Facts labels included in this section are clear, relevant, and enhance the educational value of the content. [e]	
The SMART Goals overview is thorough and easy to follow. [e]	
The explanation of how to develop and implement SMART Goals is clear and practical. [e]	
The student engagement strategies are clearly described and easy to understand. [f]	
The strategies appear feasible to implement within a school setting. [f]	
The strategies are realistic and achievable given typical school resources. [f]	
Please describe any components of "Part II. Program Design & Implementation" (pages 9-27) of the E-Guide that were difficult to follow or could benefit from additional clarification.	Open-Ended

Is there any additional information or content you think would strengthen "Part II. Program Design & Implementation" (pages 9-27) of the E-Guide? Please explain below.	
Which aspects of "Part II. Program Design & Implementation" (pages 9-27) of the E-Guide were most helpful or useful to you, and why?	
**Notes:** Questions address the following subcategories assessed: [a] Program Implementation; [b] Implementation Steps; [c] HEALTHY in Action; [d] Supplies and Equipment; [e] Education Overview; [f] Student Engagement Strategies	

Table S4. *Section Four: Survey Items Utilized in an Expert Review Study Assessing the Feasibility and Acceptability of a Smoothie-Based Nutrition Education Implementation Toolkit.*

Survey Items	Response Options
The adaptation strategies are clearly described and easy to follow. [a]	(1) Strongly Disagree; (2) Somewhat Disagree; (3) Neither Agree nor Disagree; (4) Somewhat Disagree; (5) Strongly Agree
The measurements and scaling information provided are appropriate and useful. [a]	
The adaptation strategies appear realistic and achievable within school settings. [a]	
The guidance on adapting and customizing the program for different contexts is adequate and practical. [a]	
The USDA Foods Material ID Codes included in the recipes are useful for menu planning and procurement. [a]	
The educational resources are easy to access and apply in practice. [b]	
The materials are relevant and developmentally appropriate for middle school audiences. [b]	
The resources align well with the overall goals of the HEALTHY program. [b]	
The evaluation tools are clearly described and easy to understand. [c]	
The evaluation tools appear feasible for schools to implement. [c]	
The evaluation tools appropriately align with the program's objectives and intended outcomes. [c]	
The additional resources are relevant and add value to the E-Guide. [d]	
The section provides clear and sufficient guidance for accessing these resources. [d]	
The additional resources effectively complement and enhance the E-Guide content. [d]	
Please describe any components of "Part III. Tools & Resources" (pages 28-36) of the E-Guide that were difficult to follow or could benefit from additional clarification.	Open-Ended
Is there any additional information or content you think would strengthen "Part III. Tools & Resources" (pages 28-36) of the E-Guide. Please explain below.	
Which aspects of "Part III. Tools & Resources" (pages 28-36) of the E-Guide were most helpful or useful to you, and why?	
**Notes:** Questions address the following subcategories assessed: [a] Adaptation Strategy; [b] HEALTHY Educational Resources; [c] HEALTHY Evaluation Resources; [d] Additional Resources	

Table S5. *Section Five: Survey Items Utilized in an Expert Review Study Assessing the Feasibility and Acceptability of a Smoothie-Based Nutrition Education Implementation Toolkit.*

Survey Items	Response Options
Improve fruit intake among students.	(1) Strongly Disagree; (2) Somewhat Disagree; (3) Neither Agree nor Disagree; (4) Somewhat Disagree; (5) Strongly Agree
Promote the delivery of engaging nutrition education in schools.	
Increase students' knowledge and awareness of nutrition.	
Support school nutrition staff with practical tools for implementation.	
Enhance collaboration between educators and nutrition professionals.	
On a scale from 0 to 10, how feasible would it be to use the E-Guide in practice? (0 = not at all, 10 = extremely)	Sliding Scale Ranging from 0-10
After reviewing the E-Guide, how confident do you feel that you could implement the program? (0 = not at all, 10 = extremely)	
What aspects of the E-Guide overall did you find most helpful or effective?	Open-Ended
What aspects of the E-Guide overall could be improved or clarified?	
What potential barriers to implementation do you foresee?	
Is there anything missing from the E-Guide that you feel should be included?	
Do you have any additional comments or suggestions about the E-Guide?	
